# Impact of comprehensive family history and genetic analysis in the multidisciplinary pancreatic tumor clinic setting

**DOI:** 10.1002/cam4.5059

**Published:** 2022-07-30

**Authors:** Jessica N. Everett, Shenin A. Sanoba, Xiaohong Jing, Cody Stender, Madeleine Schmitter, Ariele Baptiste, Jennifer Chun, Emily A. Kawaler, Lauren G. Khanna, Seth A. Gross, Tamas A. Gonda, Nina Beri, Paul E. Oberstein, Diane M. Simeone

**Affiliations:** ^1^ Department of Medicine New York University Langone Health New York City New York USA; ^2^ Department of Surgery New York University Langone Health New York City New York USA; ^3^ Perlmutter Cancer Center New York University Langone Health New York City New York USA; ^4^ Applied Bioinformatics Laboratories New York University Langone Health New York City New York USA; ^5^ Department of Pathology New York University Langone Health New York City New York USA

**Keywords:** family history, genetic counseling, genetic testing, germline variants, multidisciplinary care, pancreatic cancer

## Abstract

**Background:**

Genetic testing is recommended for all pancreatic ductal adenocarcinoma (PDAC) patients. Prior research demonstrates that multidisciplinary pancreatic cancer clinics (MDPCs) improve treatment‐ and survival‐related outcomes for PDAC patients. However, limited information exists regarding the utility of integrated genetics in the MDPC setting. We hypothesized that incorporating genetics in an MDPC serving both PDAC patients and high‐risk individuals (HRI) could: (1) improve compliance with guideline‐based genetic testing for PDAC patients, and (2) optimize HRI identification and PDAC surveillance participation to improve early detection and survival.

**Methods:**

Demographics, genetic testing results, and pedigrees were reviewed for PDAC patients and HRI at one institution over 45 months. Genetic testing analyzed 16 PDAC‐associated genes at minimum.

**Results:**

Overall, 969 MDPC subjects were evaluated during the study period; another 56 PDAC patients were seen outside the MDPC. Among 425 MDPC PDAC patients, 333 (78.4%) completed genetic testing; 29 (8.7%) carried a PDAC‐related pathogenic germline variant (PGV). Additionally, 32 (9.6%) met familial pancreatic cancer (FPC) criteria. These PDAC patients had 191 relatives eligible for surveillance or genetic testing. Only 2/56 (3.6%) non‐MDPC PDAC patients completed genetic testing (*p* < 0.01). Among 544 HRI, 253 (46.5%) had a known PGV or a designation of FPC, and were eligible for surveillance at baseline; of the remainder, 15/291 (5.2%) were eligible following genetic testing and PGV identification.

**Conclusion:**

Integrating genetics into the multidisciplinary setting significantly improved genetic testing compliance by reducing logistical barriers for PDAC patients, and clarified cancer risks for their relatives while conserving clinical resources. Overall, we identified 206 individuals newly eligible for surveillance or genetic testing (191 relatives of MDPC PDAC patients, and 15 HRI from this cohort), enabling continuity of care for PDAC patients and at‐risk relatives in one clinic.

## Graphical abstract

Genetic testing is recommended for all pancreatic ductal adenocarcinoma (PDAC) patients. Limited information exists regarding the utility of integrated genetics in the multidisciplinary pancreatic cancer clinic setting. We found that integrating genetics into the multidisciplinary setting significantly improved genetic testing compliance by reducing logistical barriers for PDAC patients, and clarified cancer risks for their relatives while conserving clinical resources.

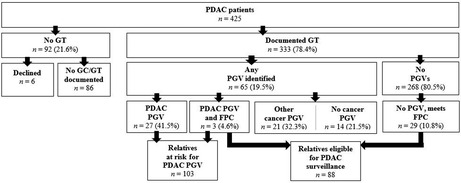

## 1 INTRODUCTION

Pancreatic ductal adenocarcinoma (PDAC) is poised to become the second leading cause of cancer‐related deaths in the United States by 2030, with disease incidence increasing and no meaningful change in mortality.^1^ The majority of patients (85%–90%) are diagnosed with advanced disease, and the corresponding 5‐year survival rate is less than 5%.^2^ In the 10%–15% of patients diagnosed with disease that can be surgically treated, the 5‐year survival rate approaches 40%,^3^ but can be as high as 60%–70% for tumors less than 1 cm in size.^4^ For this reason, early detection through screening remains a critical goal in improving survival and ultimately reducing mortality from PDAC.

Recent guidelines from the National Comprehensive Cancer Network and the American Society of Clinical Oncology recommend genetic counseling and consideration of genetic testing for all patients with newly diagnosed PDAC, regardless of family history.^5^, ^6^ It has been reported that up to 10% of PDAC patients carry a gene linked to high or moderate penetrance cancer risk.^7^, ^8^, ^9^, ^10^, ^11^ Identification of a pathogenic germline variant (PGV) in one of these known susceptibility genes can have relevance for treatment of the patient with PDAC,^12^ and may represent a sentinel diagnosis for recognizing inherited syndromes with relevance to family members,^13^ including those who may benefit from PDAC surveillance.

PDAC has a relatively low incidence in the general population, making currently available screening techniques insufficiently sensitive or specific for use outside of defined high‐risk groups.^14^ The International Cancer of the Pancreas Screening consortium (CAPS), American College of Gastroenterology, and National Comprehensive Cancer Network (NCCN) have published guideline recommendations for surveillance in individuals meeting specific high‐risk criteria (Table 1).^15^, ^16^, ^17^ Surveillance in these groups includes use of MRI/MRCP and endoscopic ultrasound (EUS), with one of these tests performed annually, although without consensus on the preferred modality or value of alternating their use.^15^ Several published studies on surveillance outcomes in small cohorts of patients meeting published criteria show promise in increasing detection of surgically resectable PDAC, with one study demonstrating a 5‐year survival rate of 60%.^18^, ^19^, ^20^, ^21^ Additionally, in the most recent surveillance study of individuals meeting published high‐risk criteria, all PDAC diagnoses ultimately occurred in PGV carriers.^22^ Such studies highlight that optimization of PDAC surveillance efforts and outcomes will require complete evaluation of genetic status in families.

**TABLE 1:** High risk criteria for recommended PDAC surveillance

	Criteria	Age
1	Two or more relatives with PDAC on same side of family, where 2 affected are first degree related to each other, and at least 1 affected is first degree related to subject	50, or 10 years younger than earliest diagnosis of PDAC in family
2	Pathogenic or likely pathogenic variant in any of the following genes: *ATM*, *BRCA1*, *BRCA2*, *MLH1*, *MSH2*, *MSH6*, *PALB2*, *PMS2*, AND a first or second degree relative with PDAC	50, or 10 years younger than earliest diagnosis of PDAC in family
3	Pathogenic or likely pathogenic variant in *CDKN2A*	40 or older
4	Peutz‐Jeghers syndrome with *STK11* pathogenic or likely pathogenic variant	35 or older
5	Hereditary pancreatitis with *PRSS1* pathogenic or likely pathogenic variant and history of pancreatitis	40 or older

Abbreviation: PDAC, pancreatic ductal adenocarcinoma.

Multidisciplinary clinics are increasingly recognized as the optimal approach to providing coordinated oncology care, leading to changes in recommendations, improved access to specialists, and shorter time to initial treatment in PDAC patients.^23^, ^24^, ^25^ Genetic services are a well‐established part of high quality multidisciplinary care for breast cancer, where genetic testing for select patients has had recognized relevance for surgical decision‐making for nearly two decades.^26^ With the Society of Gynecologic Oncology's 2014 recommendation that all women with ovarian, fallopian tube, and peritoneal carcinoma be offered genetic counseling and testing, multidisciplinary gynecologic oncology clinics have also worked to find ways to deliver genetic services efficiently. Various genetic service delivery approaches have been utilized, with the highest rate of uptake noted in “mainstreaming” of services with oncology team members providing direct access to testing, followed by post‐test genetic counseling involvement to discuss results.^27^, ^28^, ^29^ Multidisciplinary clinics can also meet the needs of individuals at increased risk for cancer based on family history or gene status, providing a resource for personalized risk assessment and management planning, updated information over time, and access to participation in research registries and clinical trials to drive translational research.^30^

While prior studies have commented on the treatment and survival‐related outcomes for PDAC patients in multidisciplinary clinics, limited information exists regarding the impact of genetic counseling and testing services in this setting. Here, we specifically explored the uptake and outcomes of integrated genetic services in a multidisciplinary clinic focused on PDAC patients and high‐risk individuals, and hypothesized that this integration could improve compliance with guideline‐based genetic testing, and lead to changes in clinical recommendations with significant benefit for patients and families.

## 2 METHODS

Study subjects included PDAC patients and high‐risk individuals (HRI), the latter defined as unaffected individuals at increased risk for PDAC due to any family history of PDAC and/or PGV status, evaluated between July 2017 and March 2021. One group of PDAC patients was evaluated in the multidisciplinary pancreatic tumor clinic (MDPC) at NYU Langone Health by a team of care providers including gastroenterologists, genetic counselors, medical oncologists, and hepato‐pancreato‐biliary surgeons for coordinated care management. A second group of PDAC patients receiving treatment at the same institution, but outside of the MDPC structure, was also reviewed as a comparison group. Data were reviewed for all HRI and PDAC patients seen via the MDPC, and for PDAC patients receiving clinical care outside of the MDPC during the study timeframe. The data were de‐identified for analysis of the following variables: patient age, sex, race/ethnicity, date of service, genetic service delivery model utilized (see below), genetic test results, and family history of PDAC. The study was undertaken as a quality assurance initiative, and was reviewed with the institutional IRB which determined the study did not meet criteria for human subject research and was exempt from full IRB review. There was no patient or public involvement in the design of this quality initiative.

## 3 GENETIC COUNSELING AND TESTING

PDAC patients evaluated through the MDPC accessed genetic testing via a “standard model” of pre‐test genetic counseling, or via a “mainstream model” of testing ordered directly by their oncology team, followed by post‐test result disclosure by a genetic counselor. The method of genetic service delivery utilized was dependent on physician discretion, prioritizing patient preference and convenience. Patients treated outside the MDPC structure had access to standard genetic counseling via referral by their treating oncologist. All HRI accessed testing via the standard model. Three‐generation cancer family pedigrees were generated for all patients seen via the standard model. For PDAC patients tested through the mainstream model, family history was reviewed by the oncology team for cancer diagnoses in first‐degree relatives at a minimum. PDAC patients found to have a PGV or first‐degree relative with PDAC were contacted when possible to obtain a full three generation cancer pedigree.

Genetic testing offered to new MDPC subjects evaluated via either model ranged from panels of 16–91 genes, but all included sequencing and large rearrangement analysis for a minimum set of the following genes: *APC*, *ATM*, *BMPR1A*, *BRCA1*, *BRCA2*, *CDKN2A*, *EPCAM*, *MLH1*, *MSH2*, *MSH6*, *PALB2*, *PMS2*, *SMAD4*, *STK11*, *TP53*, and *VHL*. Results for PDAC patients tested outside of the MDPC were reviewed and PGVs counted for each group. PGVs relevant for current pancreas surveillance guidelines (*ATM*, *BRCA1*, *BRCA2*, *CDKN2A*, *MLH1*, *MSH2*, *MSH6*, *PALB2*, *PMS2*, *PRSS1*, *STK11*) (Table 1) were categorized as PDAC PGVs for the purposes of quantifying relatives with PDAC risk.

### 3.1 Statistical analyses

Statistical analyses were used to evaluate the data distribution of variables between the patients with PDAC and HRI. Statistical analyses included Pearson's chi‐square, Pearson's chi‐square with Yates continuity correction, Welch two sample t test with a significance level of *α* = 0.05, and two‐proportion *Z* test. All analyses were completed using R version 4.1.1.

## 4 RESULTS

A total of 969 subjects were evaluated through the MDPC, including 544 HRI (56.1%) and 425 with PDAC (43.9%). An additional 56 PDAC patients were treated at the same institution, but outside of the MDPC structure. Demographics for the study population are shown in Table 2. HRI were significantly more likely to be white, female, and of Ashkenazi Jewish ancestry than MDPC PDAC patients (*p* < 0.01 for all variables). The average age of HRI was also significantly younger than MDPC PDAC patients (55.8 years vs 66.9 years; *p* < 0.01), as expected given age‐based PDAC surveillance guidelines, with imaging typically initiated at age 50. PDAC patients seen in the MDPC were significantly more likely to be white than PDAC patients seen outside the MDPC (*p* < 0.01), but there were no other significant differences in demographic variables for these groups.

**TABLE 2:** Demographics of patients evaluated in the multidisciplinary pancreas clinic

	MDPC	Non‐MDPC PDAC (*n* = 56)	High‐risk individuals (*n* = 544)
	PDAC (*n* = 425)		
Sex (*n*, %)			
Male	188 (44.2)	32 (57.1)	190 (34.9)
Female	237 (55.8)	24 (42.9)	354 (65.1)
Mean age (years, range)	66.9 (35–93)	67.1 (47–84)	55.8 (26–84)
Race (*n*, %)			
White	288 (67.8)*	26 (46.4)	491 (90.3)**
Black	39 (9.2)	10 (17.9)	18 (3.3)
Hispanic	53 (12.5)	11 (19.6)	16 (2.9)
Asian	31 (7.3)	9 (16.1)	19 (3.5)
Other	14 (3.3)	0	0
Ashkenazi Jewish ancestry (*n*, %)			
Yes	83 (19.5)	3 (5.4)	204 (37.5)**
No	342 (80.5)	53 (94.6)	340 (62.5)

* *p* < 0.01, MDPC PDAC versus Non‐MDPC PDAC, for all demographic variables.

** *p* < 0.01 HRI versus MDPC PDAC.

### 4.1 Genetic testing and outcomes in PDAC patients

**FIGURE 1:**
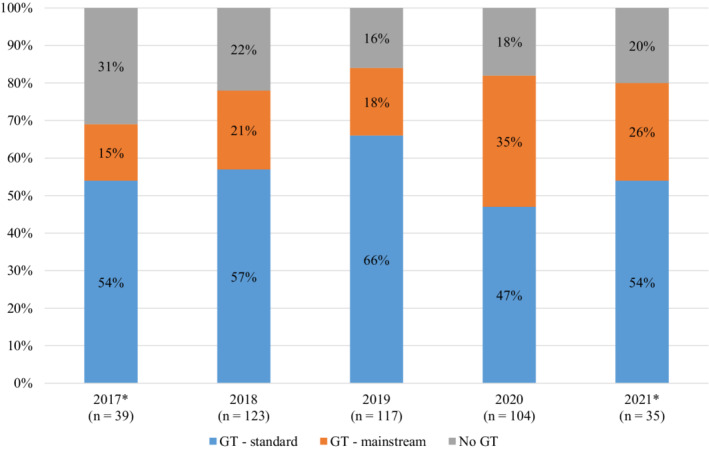
Completed genetic testing by service delivery method for all MDPC PDAC patients. GT, genetic testing. *Partial data available for these years; clinic opened in July 2017, study end date in March 2021.

Of the 425 patients with PDAC seen in the MDPC during the study period, 333 (78.4%) had documentation of completed genetic testing available, and 92 (21.6%) did not (declined, *n* = 6; no record of genetic testing, *n* = 86). The rate of completed genetic testing improved over time, from 69% in the first year to 80% in the last year of the study period (Figure 1). The majority of tested patients (236/333, 70.9%) had testing through the standard genetic counseling model, but the proportion of patients accessing testing through the mainstream model increased over time, from 15% of total patients in the first year to 26% in the last year of the study period (Figure 1). Review of records for 56 PDAC patients treated at the same institution during the study period outside of the MDPC showed that only 2/56 (3.6%) had documentation of completed genetic testing, a significant difference from the MDPC group (*p* < 0.01).

**FIGURE 2:**
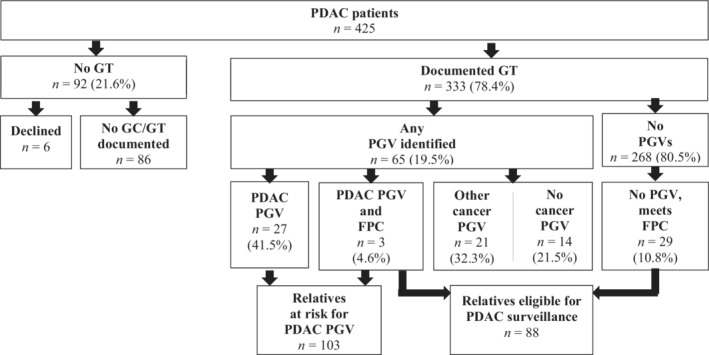
Genetic testing and outcomes for PDAC patients evaluated in MDPC. FPC, familial pancreatic cancer; GC, genetic counseling; GT, genetic testing; PDAC, pancreatic ductal adenocarcinoma; PGV, pathogenic germline variant.

Of the 333 MDPC patients with PDAC and documented germline testing, 65 patients (19.5%) had at least one PGV, with 69 PGVs identified in 19 genes (Figure 2, Table S1). Among the 65 PGV carriers, 30 (46.2%) had PDAC PGVs; this included 27 patients with no family history of PDAC, as well as three patients who also had a first‐degree relative with PDAC, resulting in a concurrent diagnosis of familial pancreatic cancer (FPC). Collectively, these 30 PDAC PGV carriers had 103 first‐degree relatives at 50% risk for the PDAC PGV, all potentially eligible for surveillance dependent on their genetic test results. For 23/30 PDAC patients, the identified PGVs had relevance for approved treatment options for their own cancer diagnosis (3 *BRCA1*, 17 *BRCA2*, 2 *MSH6*, 1 *PMS2*).^12^, ^31^, ^32^ An additional 35 PDAC patients had other PGVs identified with no known link to PDAC risk, including 21 patients found to carry PGVs with other cancer risks associated. Overall test results in 48/65 (73.9%) PDAC patients (Table S1) had relevance for other cancer screening or risk reducing options for families, including breast, colorectal, melanoma, ovarian, prostate, and endometrial cancers.

The remaining 268 (80.5%) PDAC patients had no PGVs identified. In review of family history, 29/268 (10.8%) PDAC patients with no PGVs had a personal and family history of PDAC that met FPC criteria. Together with the 3 FPC families identified among PGV carriers, these 32 newly‐identified FPC patients had 88 first‐degree relatives newly eligible for PDAC surveillance. Fourteen of these 32 PDAC patients with FPC (43.8%) had at least one first‐degree relative present to the MDPC for their own risk evaluation; these relatives are among the 544 HRI described.

### 4.2 Genetic testing and outcomes for high risk individuals

**FIGURE 3:**
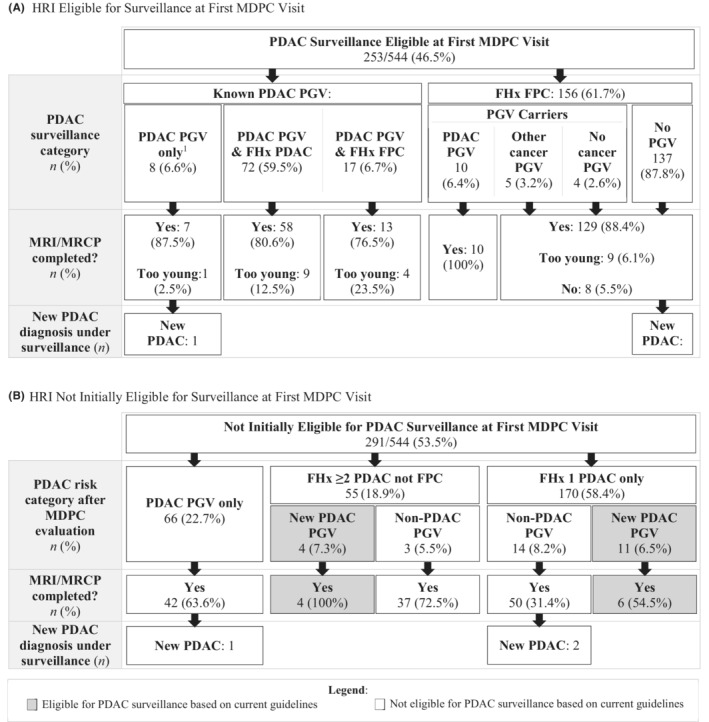
(A) Genetic testing and outcomes for 544 high risk individuals evaluated in the MDPC. (A) HRI eligible for surveillance at first MDPC visit. ^1^Eligible for surveillance based on PGV alone: *CDKN2A*, *n* = 7; *PRSS1*, *n* = 1. (B) HRI not initially eligible for survelliance at first MDPC visit. FHx, family history. FPC, familial pancreatic cancer; GT, genetic testing; HRI, high risk individual; PDAC, pancreatic ductal adenocarcinoma; PGV, pathogenic germline variant.

A total of 544 HRI from 480 families were seen via the MDPC during the study time frame, and all received standard model genetic counseling during their visit. Out of these 544 HRI, 253 (46.5%) were eligible for pancreatic surveillance at presentation to the MDPC due to known PGV carrier status (*n* = 8), known PGV with family history of PDAC (*n* = 72), known PGV with family history of FPC (*n* = 17), or family history meeting FPC criteria (*n* = 156) (Figure 3). New PDAC diagnoses occurred in two HRI who met PDAC surveillance guidelines at baseline (one with a known *CDKN2A* PGV and family history of PDAC; one with FPC family history and negative genetic testing via the MDPC). These new PDAC diagnoses led to changes in surveillance eligibility for two family members of these HRIs. Genetic testing was completed for the remaining 156 HRI meeting FPC criteria, with PGVs identified in 19 (12.2%), including 10 (6.4%) with PDAC PGVs, and 5 with PGVs linked to other cancer risks. Among the 19 FPC family HRI PGV carriers, 15 (78.9%) PGVs (Table S2) had relevance for other cancer screening or risk‐reducing options, including breast, ovarian, and prostate cancer. Additionally, one patient was diagnosed with an early‐stage breast cancer based on follow‐up for a *BRCA1* PGV identified through genetic testing in the MDPC.

Of the 544 total HRI, 291 (53.5%) did not meet published criteria for PDAC surveillance at their first MDPC visit (Figure 3B). This group included 66 (22.7%) with a known PDAC PGV (54 *BRCA2*, 5 *PALB2*, 5 *ATM*, 2 *BRCA1*) previously identified outside the MDPC on the basis of personal or family history of other cancer diagnoses, but no known PDAC family history. MRI/MRCP was completed in 42 (63.6%) of these subjects, and one new PDAC was diagnosed in a *BRCA2* carrier with no family history of PDAC from this group. Another 55 subjects (18.9%) presented based on having ≥2 relatives with PDAC not meeting FPC criteria. Genetic testing led to identification of new PDAC PGVs in 4/55 (7.3%) confirming new eligibility for PDAC surveillance; 3 (5.5%) non‐PDAC PGVs were also identified. All four new PDAC PGV carriers completed an MRI, as did 37 subjects with no relevant PGV finding; no cancers have been identified in this group. Finally, 170/291 subjects (58.4%) presented with a single relative with PDAC, including 25 referred primarily to discuss management of a known pancreatic cystic neoplasm who were then identified to have family history of PDAC at their MDPC visit. In this group, 11/170 (6.5%) tested positive for a PDAC PGV, including eight found to carry a known PGV previously identified in a relative; these HRI were subsequently newly eligible for PDAC surveillance. Another 14/151 (9.3%) had a non‐PDAC PGV identified, while 145 (85.3%) had no PGV found. Two new PDACs were diagnosed in subjects with a single affected relative and no PGV, including one in a patient who presented with an incidental pancreatic cyst.

HRI who remained in the ineligible group sought information regarding risk and management options, and had a discussion of the potential costs, benefits, and risks of surveillance with the MDPC team. These HRI were also offered the option of annual clinical follow up for ongoing discussion of individualized management in the context of changes in available data, family history or personal risk factors, and surveillance guidelines over time. All HRI not engaged in surveillance were encouraged to remain in contact with the MDPC with any future changes to their personal or family cancer history that might impact screening eligibility.

## 5 DISCUSSION

The evolving guidelines for genetic counseling and testing in patients and families with PDAC create new opportunities to identify individuals at increased risk, but clinical and logistical barriers to maximizing utilization of testing remain. Here, we describe an improved overarching approach to address unmet needs in a multidisciplinary setting. Overall, integration of genetic services into the MDPC for PDAC patients and HRI created a unified and streamlined clinical setting, and led to several beneficial outcomes. Incorporating genetic counseling and/or genetic testing as part of an MDPC visit for PDAC patients reduced the overall burden of genetic testing for patients and their HR relatives, as well as for oncology providers. Through a combination of standard model genetic counseling and testing at the time of MDPC visit, as well as a mainstream model of testing ordered by oncology staff, compliance with recommended genetic testing for PDAC patients reached 80% by the end of the study period. For comparison, genetic testing was completed by only 2/56 (3.6%) PDAC patients treated at the same institution, but outside the MDPC structure (i.e., without integrated genetics services). One study prior to universal testing recommendations for PDAC patients found that the typical process of referral for a separate visit for genetic services led to 32% of PDAC patients referred to genetics, and only 19% follow through due to several barriers, including worsening disease severity, insurance concerns, and travel challenges.^33^ With the arrival of the recommendations for genetic testing in all PDAC patients, alternative models of service delivery have been suggested to maximize testing completion, and evaluated under research study conditions. Embedding a genetic counselor into the oncology clinic for direct contact with all PDAC patients led to high study participation (87%) and uptake of genetic testing in 97% of study participants,^34^ but this model may not be feasible in all oncology clinic settings. Mainstream models under study conditions have achieved genetic test completion rates of 65%–71%.^29^, ^35^ Our results demonstrate clinical implementation of genetic service delivery outside of research study conditions reaching a completion rate of 80%. Both types of service delivery, standard and mainstream, were utilized successfully in the clinic and the ability to access both models maximized use of available resources, while also reducing the burdens on patients and families and allowing providers to overcome challenges directly. Our experience adds to the growing body of literature in favor of facilitating point of care genetic services within a multidisciplinary or oncology clinic setting, rather than requiring separate visits. The optimal workflow for implementation of genetic counseling and testing within these settings will likely vary based on available resources and specific clinic and patient priorities. Clinics wanting to introduce these services can adapt to utilize the approach or approaches that best fit within their existing structure.

The MDPC structure also facilitated team communication and removed the barrier of dependency on provider referral for completion of testing. The entire MDPC care team had direct access to genetic counselors to discuss genetic testing results and patient questions as they arose. Reminders to obtain genetic testing in PDAC patients and review of genetic test results and PGV outcomes was also incorporated into weekly tumor board case presentations. This created an additional opportunity for team members to increase awareness of guidelines for genetic testing, identify patients who may still need testing, and further reduce the burden on specific treating physicians to make referrals and follow up on outcomes.

Provision of care through an MDPC structure that can accommodate *both* PDAC patients and HRI also led to more efficient use of testing resources and more accurate risk information for relatives. PDAC PGVs were identified in 8.7% of PDAC patients in our population, consistent with previous studies.^7^, ^8^, ^9^, ^10^, ^11^ In addition to providing information relevant for treatment and clinical trial options, identifying PGVs in patients with PDAC clarified risks for their relatives and created a seamless path to resources for genetic counseling, genetic testing, and PDAC surveillance through the same clinical mechanism. Among the 32 probands with PDAC meeting FPC criteria, 14 (43.8%) had at least one first‐degree relative present for screening through the MDPC, and all of these relatives had prior contact with the clinic staff during the course of the PDAC patient's care. For PDAC patients with no PGV identified and no family history of PDAC, their own testing alleviated the need for potentially unnecessary downstream testing of unaffected relatives. Nearly one‐third of the HRI evaluated in our clinic presented on the basis of a single relative with PDAC who died without completing genetic testing. Qualitative research has found that fear of personal risk for PDAC is a common concern for these individuals.^36^ Even when genetic testing is offered and no PGV is identified, risk assessment for these HRI is limited by the absence of genetic information for their single affected family member. Thus, continued efforts to increase testing compliance in individuals with PDAC can help address this information gap for families.

Genetic counseling and testing in HRI clarifies risk for families in several ways. Until recently, the cost of genetic testing and its limited use in PDAC meant that many families who met FPC criteria and engaged in PDAC surveillance had no genetic testing, or testing for a limited number of genes. Now, the ability to identify an underlying PDAC PGV in some FPC families via comprehensive genetic testing ultimately enables the sharing of gene‐specific data for PDAC risk, as well as recommendations for screening for other PGV‐related cancers. Furthermore, results from a recent study found that all of the PDAC diagnoses made under surveillance occurred in PGV carriers.^22^ Long‐term data regarding PGV carriers is crucial to collect, as estimations for gene‐specific PDAC risk continue to change. There is a clear need for evidence‐based recommendations for PGV carriers, especially as most PGV carriers who develop PDAC will be the first in their family to do so. Overall, it is increasingly apparent that complete genetic characterization of FPC families will be critical for understanding the natural history of PDAC progression.

While identification of PDAC PGVs was most relevant to determining pancreatic cancer surveillance eligibility in families, it is important to note that additional PGVs with relevance for cancer screening or family planning were also identified, and that the overall positive rate was 19.5% in our study inclusive of all PGV findings. PGV identification rate varies across studies of PDAC patients, partially due to differences in the testing performed. Among PDAC patients tested with broader panels, PGV rates of 17%–33% have been reported when testing includes genes with no currently‐established PDAC risks.^34^, ^35^ Evidence to establish and refine which genes are definitively associated with increased PDAC risk continues to accumulate over time. Indeed, while PGVs in certain DNA damage repair genes (e.g. *BRIP1*, *CHEK2*, *FANCC*, *NBN*, *RAD50*, *RAD51C*, *RAD51D*) are not currently linked to PDAC risk or included in PDAC surveillance guidelines, there is considerable interest in defining their role in PDAC tumorigenesis and treatment response.^8^, ^37^, ^38^ As broader multi‐gene panels become more common, documenting all findings in pancreatic cancer families through registries will be important to advancing understanding of the missing heritability for pancreatic cancer, and also offers opportunities to provide couseling for other cancer risks.

More than half of HRI presenting to the MDPC did not meet existing criteria for PDAC surveillance; similarly, an Australian study found that 50% of inquiries about PDAC screening came from HRI who did not meet criteria.^39^ These parallel findings highlight the need to recognize the viable concerns of this group of individuals, whose informational and counseling needs are best addressed by a healthcare team with appropriate knowledge and expertise. In our group of 291 HRI not meeting criteria at their first MDPC visit, 15 became eligible for PDAC surveillance after genetic counseling and testing. Another 276 were able to discuss fully the risks, benefits, and limitations of current PDAC surveillance options, along with their specific personal and familial risk factors, to make informed decisions about their care. Importantly, these HRI were also invited to participate in a research registry (NCT04970056; www.precedestudy.org) to follow their screening decisions and outcomes longitudinally, which will provide the evidence base that enables guidelines to evolve over time.

Through risk assessment, genetic testing, and subsequent cancer screening coordinated through the MDPC, six individuals were diagnosed with cancers, including five new PDAC diagnoses and one breast cancer. For individuals found to carry cancer risk PGVs through the MDPC, availability of genetic services within the clinic allows seamless referral for non‐PDAC cancer screening, along with compliance checks as part of the routine MDPC follow up. The genetics team within the MDPC also utilizes the registry to monitor compliance with annual PDAC screening follow up and completion. This registry also provides opportunities to maintain contact with individuals who do not meet current eligibilitly critiera for PDAC screening as knowledge and recommendations evolve. Three of the five PDAC diagnoses in our study occurred in subjects who *did not* meet surveillance criteria; these diagnoses had important screening implications for the families involved, highlighting the importance of registry follow‐up for all subjects who present with concern about PDAC risk. Our experiences demonstrate the potential short‐ and long‐term benefits of inclusive genetic services in the multi‐disciplinary clinical setting.

The study has some limitations. Calculation of genetic testing uptake for PDAC patients was based on documentation of completed genetic testing, and we were not able to determine how many patients declined genetic testing when offered by their oncology team, or how many were never offered genetic services. The genetic panel used for testing varied across subjects during the study period. However, all subjects tested through the MDPC had a multi‐gene panel including a minimum set of 16 genes with known PDAC risks and associated surveillance guidelines. Standardization of genetic testing panels needed for PDAC patients and HRI will be important in future research studies. Finally, while we are able to report on the number of relatives of PDAC probands who presented for care under the same MDPC structure at our institution, we may have missed some information on relatives who pursued screening or genetic testing at outside institutions, despite our inquiries.

In conclusion, a multidisciplinary clinic model that incorporated genetic services directly into the care of patients with PDAC and HRI led to benefits for patients, family members, and healthcare providers. Compliance with recommended genetic testing for PDAC patients was significantly improved through the MDPC model, leading to more efficient use of testing resources and less unnecessary downstream testing of unaffected relatives. The MDPC structure also facilitated team communication, increasing engagement and involvement of oncology providers in the genetic counseling and testing process. The MDPC also reduced logistical barriers for the relatives of PDAC patients, who could coordinate their own follow‐up care in the same clinical setting. Genetic counseling and testing in the MDPC clarified cancer risks for families who met criteria for PDAC surveillance, and provided a resource for all patients seeking individualized information about personal cancer risk and management options.

## AUTHOR CONTRIBUTIONS

Jessica N. Everett: conceptualization, data curation, methodology, project administration, investigation, writing—original draft, writing—review and editing. Shenin A. Sanoba: data curation, methodology, visualization, writing—original draft, writing—review and editing. Xiaohong Jing: data curation, writing—review and editing. Cody Stender: data curation, visualization, writing—review and editing. Madeleine Schmitter: data curation, visualization, writing—review and editing. Arele Baptiste: data curation. Jennifer Chun: project administration, writing—review and editing. Emily A. Kawaler: formal analysis. Lauren G. Khanna: resources, supervision, writing—review and editing. Seth A. Gross: resources, supervision, writing—review and editing. Tamas A. Gonda: resources, supervision, writing—review and editing. Nina Beri: resources, supervision, writing—review and editing. Paul E. Oberstein: resources, supervision, writing—review and editing. Diane M. Simeone: conceptualization, project administration, methodology, resources, supervision, writing—review and editing.

## FUNDING INFORMATION

Funding for this work was provided by Project Purple, Trovanow, and the Alliance of Families Fighting Pancreatic Cancer.

## CONFLICT OF INTEREST

The authors declare no conflicts of interest.

## ETHICAL APPROVAL

The study was undertaken as a quality assurance initiative, and did not meet criteria for human subject research requiring IRB review.

## Supporting information


Table S1

## Data Availability

The data that support the findings of this study are available from the corresponding author upon reasonable request.
