# Supplementary material for: Impact of comprehensive family history and genetic analysis in the multidisciplinary pancreatic tumor clinic setting
Source: Cancer Med. 2022 Jul 30;12(3):2345–55. doi: 10.1002/cam4.5059 (PMC9939217; doi:10.1002/cam4.5059)
Supplement: Supplementary file 1 — Table S1 [file CAM4-12-2345-s001.docx]

**Supplemental Table 1.** Pathogenic Germline Variants in PDAC Patients (*n*=65) Evaluated in the MDPC

|  | **Gene** | **PGVs (*n*)** | **Other Cancers with Current Screening Implications** |
| --- | --- | --- | --- |
| **Genes with PDAC Risk** | *ATM* | 6 | Breast |
|  | *BRCA1* | 3 | Breast, ovarian, prostate, cutaneous and ocular melanoma |
|  | *BRCA2* | 17 |  |
|  | *CDKN2A* | 1 | Cutaneous melanoma |
|  | *MSH6* | 2 | Colon, endometrial, ovarian, urothelial, gastric, prostate, brain, skin |
|  | *PMS2* | 1 |  |
| **Other Genes** | *APC* p.I1307K | 10 | Colon |
|  | *BRIP1* | 1 | Ovarian |
|  | *CFTR* | 9 | None |
|  | *CHEK2* | 5 | Breast, colon |
|  | *FANCC* | 3 | None |
|  | *MITF* | 1 | Cutaneous melanoma, renal |
|  | *MUTYH* | 2 | Colon (dependent on family history) |
|  | *NBN* | 3 | None |
|  | *NF1* | 1 | Breast, pheochromocytoma, malignant peripheral nerve sheath tumor |
|  | *RAD50* | 1 | None |
|  | *RAD51C* | 1 | Ovarian |
|  | *RAD51D* | 1 | Ovarian |
|  | *TP53* | 1 | Primarily breast, brain, sarcoma, adrenal, and hematological; broad spectrum of cancer types |

*n* = 68 PGVs identified total. One patient with 2 PGVs (*BRCA2* and *CFTR* heterozygote), and one patient with 3 PGVs (*CDKN2A*, *RAD50*, and *MUTYH*).

**Supplemental Table 2.** Pathogenic Germline Variants in HRI (*n*=210) Evaluated in the MDPC

|  | **Gene** | **PGVs (*n*)** | **Current Risk/Management Guidelines for Other Cancers** |
| --- | --- | --- | --- |
| **Genes with PDAC Risk** | *ATM* | 16 | Breast |
|  | *BRCA1* | 15 | Breast, ovarian, prostate, cutaneous and ocular melanoma |
|  | *BRCA2* | 129 |  |
|  | *CDKN2A* | 8 | Cutaneous melanoma |
|  | *PALB2* | 13 | Breast, ovarian |
|  | *MLH1* | 2 | Colon, endometrial, ovarian, urothelial, gastric, prostate, brain, skin |
|  | *MSH2* | 2 |  |
|  | *MSH6* | 5 |  |
|  | *PMS2* | 3 |  |
|  | *PRSS1* | 1 | No |
| **Other Genes** | *APC* p.I1307K | 4 | Colon |
|  | *CHEK2* | 5 | Breast, colon |
|  | *NF1* | 1 | Breast, pheochromocytoma, malignant peripheral nerve sheath tumor |
|  | *SPINK1* | 1 | No |
|  | *CFTR* | 7 | No |
|  | *MUTYH* | 1 | Yes, depending on family history of colon cancer |
|  | *NTHL1* | 1 | No |
|  | *RAD51D* | 1 | Yes; ovarian |

Of note*, n* = 8 HRI tested positive for a known familial PGV previously identified in a relative (*BRCA2, n* = 7; *PMS2*, *n* = 1).

*n* = 215 PGVs identified total. Five patients had 2 PGVs: *BRCA1*/*BRCA2*; *BRCA1*/*MSH6*, *BRCA1*/*ATM* (*n* = 2); *CFTR*/*SPINK1*.
